# Correction: De Conti et al. Histological Lesions and Replication Sites of PCV3 in Naturally Infected Pigs. *Animals* 2021, *11*, 1520

**DOI:** 10.3390/ani11113277

**Published:** 2021-11-16

**Authors:** Elisa Rigo De Conti, Talita Pilar Resende, Lacey Marshall-Lund, Albert Rovira, Fabio Augusto Vannucci

**Affiliations:** 1 Veterinary School, Federal University of Rio Grande do Sul, Porto Alegre 91540-000, Brazil; elisa.rdc@hotmail.com; 2 Veterinary Diagnostic Laboratory, College of Veterinary Medicine, University of Minnesota, 1333 Gortner Avenue, Saint Paul, MN 55108, USA; leml@umn.edu (L.M.-L.); rove0010@umn.edu (A.R.); 3 Department of Veterinary and Biomedical Sciences, College of Veterinary Medicine, University of Minnesota, 1971 Commonwealth Avenue, Saint Paul, MN 55108, USA; talitaresendevet@gmail.com

The authors wish to make the following correction to this paper [1]:

**Figure/Table Legends**

In the original article, there was a mistake in the legends for Figure 4 and Figure 5. The correct legends appear below. The authors apologize for any inconvenience caused and state that the scientific conclusions are unaffected. The original article has been updated.

1st paragraph, page 4:

Lymphoplasmacytic periarteritis was the most frequently observed lesion in the kidney samples and in lymph node samples (Table 1, Figure 3a). Lymphoplasmacytic periarteritis was also observed in liver and pancreas (Figure 5a) and spleen samples (Figure 4a). Other less frequent histopathological lesions are presented in Table 1.

2nd paragraph, page 4:

The other organs with PCV3-ISH-RNA positive signals were kidney (9/13), liver (9/11), spleen (10/12) (Figure 4b) and lymph node (5/6) (Figure 3b). The distribution of the PCV3-ISH-RNA positive signals (Table 3) was predominantly multifocal (72/84).

Legends:

**Figure 4**

(a) “Lymph node” needs to be replaced by “Spleen”.

(b) “Lymph node” needs to be replaced by “Spleen”.

**Figure 5**

(a) “Liver” needs to be replaced by “Pancreas”.

(b) “Liver” needs to be replaced by “Pancreas”.

The authors would like to apologize for any inconvenience to the readers caused by these errors.
