# Supplementary material for: Histological Lesions and Replication Sites of PCV3 in Naturally Infected Pigs
Source: Animals (Basel). 2021 May 24;11(6):1520. doi: 10.3390/ani11061520 (PMC8224807; doi:10.3390/ani11061520)
Supplement: Supplementary file 1 [file animals-11-01520-s001.zip › animals-1190314-proofread-suppl.pdf]

Suplmentary table 1: Summary of all cases selected for *in situ* hybridization (ISH-RNA). PCV3 and PCV2 real-time PCR results, age, histopathologic classification, and PCV3-ISH-RNA results.

Case	PCV2 PCR	Age (days)	PCV3 PCR Ct values and classification		Heart	PCV3-ISH-RNA	Lung	PCV3-ISH-RNA	Liver	PCV3-ISH-RNA	Kidney	PCV3-ISH-RNA	Spleen	PCV3-ISH-RNA	Lymph node	PCV3-ISH-RNA
1	-	Fetus	14.97	Highly positive	yes	+	no	N/A	no	N/A	yes	+	no	N/A	no	N/A
2	-	19	16.40	Highly positive	yes	+	yes	+	no	N/A	no	N/A	no	N/A	no	N/A
3	-	N/A	22.06	Moderately positive	no	N/A	no	N/A	no	N/A	no	N/A	yes	+	no	N/A
4	-	70	25.02	Moderately positive	no	N/A	yes	+	no	N/A	yes	-	no	N/A	yes	-
5	-	21	25.67	Moderately positive	yes	+	yes	+	yes	-	no	N/A	yes	+	no	N/A
6	-	21	37.78	Mildly positive	yes	+	no	N/A	no	N/A	yes	-	yes	+	no	N/A
7	+	49	23.00	Moderately positive	yes	+	yes	+	no	N/A	no	N/A	yes	+	no	N/A
8	+	N/A	39.70	Mildly positive	no	N/A	yes	-	no	N/A	yes	-	yes	-	no	N/A
9	+	35	39.29	Mildly positive	yes	+	no	N/A	no	N/A	no	N/A	no	N/A	no	N/A
10	+	Fetus	17.04	Highly positive	yes	+	yes	+	yes	+	no	N/A	no	N/A	no	N/A
11	+	Fetus	17.35	Highly positive	yes	+	yes	+	no	N/A	yes	+	no	N/A	no	N/A
12	-	Fetus	20.00	Highly positive	yes	+	yes	-	yes	+	no	N/A	no	N/A	no	N/A
13	+	7	18.04	Highly positive	yes	+	yes	+	yes	+	yes	+	yes	+	no	N/A
14	+	7	19.34	Highly positive	yes	+	yes	+	no	N/A	yes	+	yes	+	no	N/A
15	+	21	22.00	Moderately positive	yes	+	yes	+	yes	+	no	N/A	yes	+	yes	+
16	+	42	22.52	Moderately positive	yes	+	yes	+	yes	+	no	N/A	yes	+	yes	+
17	+	1	19.30	Highly positive	yes	+	yes	+	no	N/A	yes	+	no	N/A	no	N/A
18	-	Fetus	16.28	Highly positive	yes	+	yes	+	yes	+	yes	+	no	N/A	no	N/A
19	+	35	21.51	Moderately positive	yes	+	yes	+	no	N/A	no	N/A	no	N/A	no	N/A
20	+	5	23.08	Moderately positive	yes	+	yes	+	yes	+	yes	+	no	N/A	no	N/A
21	+	20	23.29	Moderately positive	yes	+	yes	+	yes	+	yes	+	yes	+	yes	+
22	+	20	26.65	Moderately positive	yes	+	yes	+	yes	+	yes	+	yes	+	yes	+
23	+	N/A	16.59	Highly positive	yes	-	yes	-	no	N/A	no	N/A	no	N/A	no	N/A
24	-	3	25.50	Moderately positive	no	N/A	yes	yes	no	N/A	no	N/A	no	N/A	no	N/A
25	-	21	27.50	Moderately positive	yes	+	yes	yes	yes	-	yes	+	yes	+	yes	+
Total	15				21	20	21	18	11	9	13	9	12	10	6	5

PCV3-ISH-RNA: a positive sign for PCV3 in *in situ* hybridization; N/A: not applied; -: negative; +: positive.
